# Integrated physiological and metabolomic analyses reveal changes during the natural senescence of *Quercus mongolica* leaves

**DOI:** 10.1371/journal.pone.0289272

**Published:** 2023-08-23

**Authors:** Yangchen Yuan, Weiqiang Zhang, Jiushuai Pang, Miaomiao Zhou, Jianying Liu, Jin Zhao, Jinming Sui, Dazhuang Huang, Minsheng Yang

**Affiliations:** 1 College of Landscape Architecture and Tourism, Agricultural University of Hebei, Baoding, Hebei, China; 2 Hongyashan State-Owned Forest Farm, Baoding, Hebei, China; 3 Meteorological Bureau of Yi County, Baoding, Hebei, China; 4 College of Forestry, Agricultural University of Hebei, Baoding, Hebei, China; Universidad Autónoma Agraria Antonio Narro, MEXICO

## Abstract

*Quercus mongolica* is a common landscape, afforestation, and construction timber species in northern China with high ecological, economic, and ornamental value. Leaf senescence is a complex process that has important implications for plant growth and development. To explore changes of metabolites during the ageing of *Quercus mongolica* leaves, we investigated physiological responses and metabolite composition in ageing leaves harvested from 15–20-year-old *Quercus mongolica*. Leaf samples of *Q*. *mongolica* were collected when they were still green (at maturity) (stage 1), during early senescence (stage 2), and during late senescence (stage 3). These leaves were then subjected to physiological index and metabolome sequencing analyses. The physiological analysis showed that the leaves of *Q*. *mongolica* changed from green to yellow during senescence, which induced significant accumulation of soluble sugar and significant reductions in the concentration of soluble protein and chlorophyll. Peroxidase and catalase were the main antioxidant enzymes mitigating leaf senescence. Metabolomic analysis identified 797 metabolites during leaf senescence. Compared to stage 1, 70 differential metabolites were screened in stage 2 and 72 were screened in stage 3. Differential metabolites in the two senescent stages were principally enriched in amino acid metabolism, lipid metabolism and secondary metabolite biosynthesis. The contents of N-oleoylethanolamine and N, N-dimethylglycine were significantly increased only in stage 2, while the contents of trifolin, astragalin, valine, isoleucine, leucine, and citric acid were significantly increased only in stage 3. Histidine, homoserine, tryptophan, tyrosine, phenylalanine, proline, norleucine, N-glycyl-L-leucine, linoleic acid, linolenic acid, gallic acid, 3-indoleacrylic acid, 3-amino-2-naphthoic acid, 3-hydroxy-3-methylpentane-1,5-dioic acid, 2,3,4-trihydroxybenzoic acid, trifolin, astragalin, DL-2-aminoadipic acid, pinoresinol dimethyl ether, dimethylmatairesinol, and lysophosphatidylcholine increased during both stage 2 and stage 3. Increasing contents of these metabolites may constitute the main mechanism by which *Q*. *mongolica* leaves adapt to senescence.

## Introduction

*Quercus mongolica* (family Fagaceae) is a broad-leaved deciduous tree distributed mainly in North and Northeast China. It is the major tree species of deciduous broad-leaved forest and coniferous broad-leaved mixed forest in the temperate zone of China [1–3]. *Quercus mongolica* is an important timber [4], sericulture [5, 6], dye, tannin [7], food, medicinal [8], brewing material and landscape species. It is also highly advantageous for creating windbreak, water conservation, and fire protection. In summary, *Q*. *mongolica* is of great economic, ornamental, and ecological value.

Leaf senescence is the final stage of leaf development. As an active physiological process, senescence includes the process of nutrient transport from senescent leaves to primary tissues, which is important for developing seeds and storage organs. Senescence has high physiological significance for nutrient recycling and seed formation in plants, as well as for plant adaptation to the environment. Leaf senescence is not merely a degenerative change; it is also a process regulated by gene expression and environmental factors at the levels of cells, organs, and tissues [9, 10]. Plants are exposed to a variety of stressful environments, and senescence constitutes an adaptive mechanism that confers resistance, thus allowing plants to complete their life cycle under stressful conditions [11, 12].

During the growth phase, leaves accumulate chemical energy and nutrients by absorbing solar energy and carbon dioxide. When they enter the senescence phase after a long period of productive photosynthesis, leaf cells undergo dramatic but orderly physiological, biochemical and metabolic changes. These changes are regulated by complex and precise physiological processes and a range of endogenous and exogenous factors [13].

For woody plants, the complex and variable cycle of seasonal change is the most important factor influencing leaf development. The gradual reduction in day length (photoperiod change) and decrease in temperature during autumn are the most important external environmental triggers of leaf senescence in deciduous trees [14–16]. At lower latitudes (25–49°N), leaf senescence is more susceptible to temperature changes [17]. Therefore, leaf senescence in *Quercus mongolica* leaves is more easily affected by temperature.

When plants are subjected to external stresses, a series of defence mechanisms regulated by antioxidant enzyme systems, osmoregulators, and cell membrane structural substances are initiated [18]. The synthesis of various functional proteins through the activation of a large number of stress-response genes and complex signalling networks results in a range of downstream physiological and metabolic products that confer tolerance to stress [19]. The most obvious phenotypic change occurring during senescence is leaf yellowing due to the decomposition of chlorophyll in chloroplasts [20]. This process is accompanied by increased oxidation and hydrolysis of macromolecules such as proteins, lipids, and nucleic acids. These metabolites play an important regulatory role in leaf senescence.

In this study, we analysed the responses of *Quercus mongolica* leaves to the natural ageing process using physiological and metabolomic methods. Our objectives were to analyse the accumulation and changes of various metabolites in plant leaves, analyse physiological changes taking place in leaves, identify differential metabolites in different growth periods; and reveal the mechanisms regulating the metabolism and growth of leaves during different periods, with the goal of providing a theoretical basis for future in-depth studies of *Q*. *mongolica*.

## Materials and methods

### Plant material

**Fig 1:**
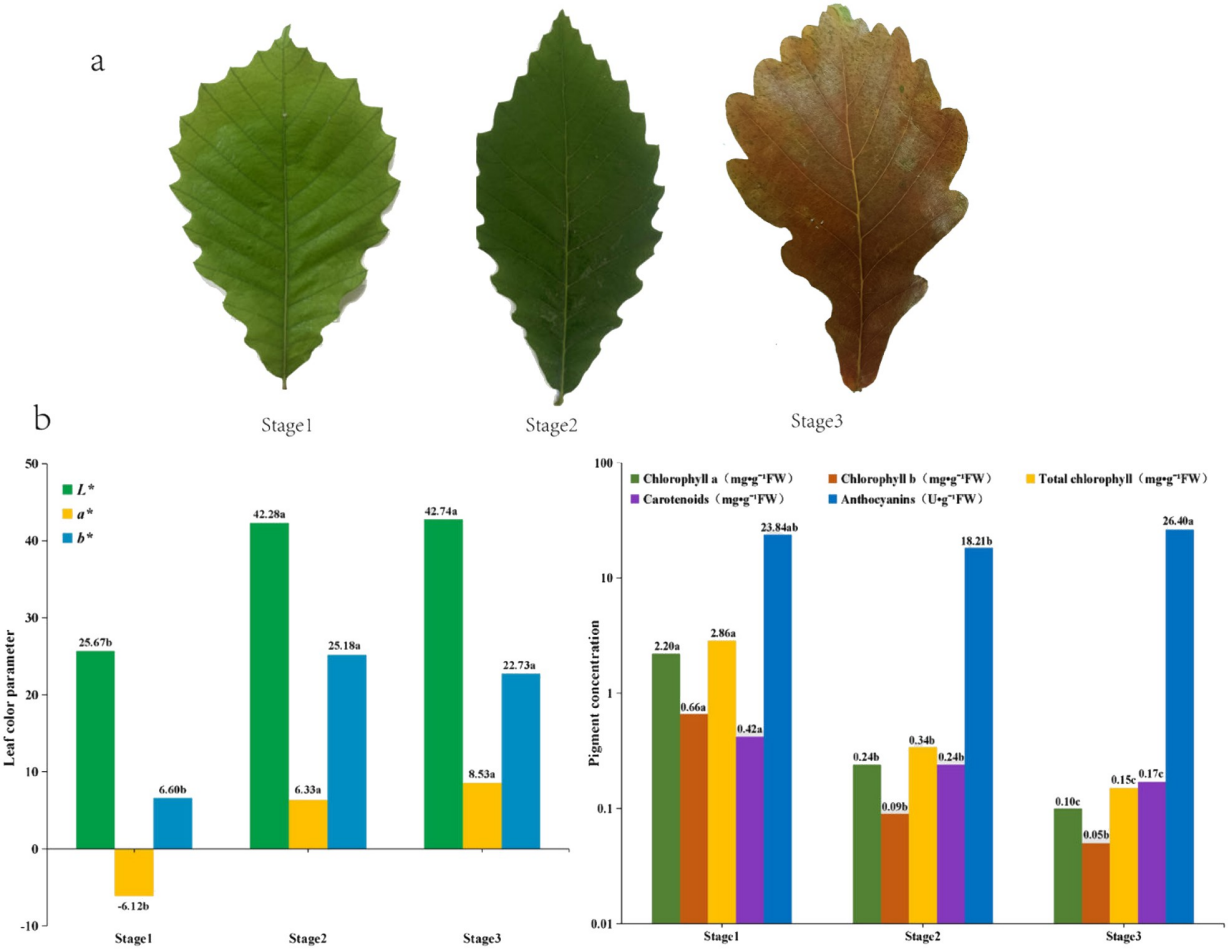
Changes in leaf colour parameters and pigment content of *Q*. *mongolica*. (a) Phenotypes of *Q*.*mongoliaca* leaves during maturity, early senescence, and late senescence. (b) Changes in leaf colour parameters. (c) Leaf pigment content and variation. Note: Different lower-case letters after the values indicate that the different groups differ significantly at the 0.05 level (same below).

The study site was a natural *Q*. *mongolica* forest located in Caijiayu (39°32′6″N, 113°52′10″E) in Yixian County, Baoding City, Hebei Province, China. Nine *Q*. *mongolica* trees with relatively consistent growth and good growth status were analysed. Three trees were assigned to each experimental treatment (total of three treatments). Leaf samples were collected during the mature (green leaf) stage (June 10), early senescence stage (in which the colours changed; September 20), and late senescence stage (in which the leaves began to fall; deciduous stage, October 20) (Fig 1a). Functional leaves in the middle of new shoots were collected from the east-, west-, south- and north-facing parts of the tree canopy. Leaf samples were immediately frozen in liquid nitrogen and stored in a refrigerator at –80°C for physiological index determination and metabolomic analysis. We denote the three periods as stages 1–3, with stage 1 serving as a control.

### Measurement of leaf colour parameters

Leaf colour parameters were analysed using a colorimeter (CR-400; Konica Minolta, Tokyo, Japan). Leaf colour was measured during three different sampling periods: maturity (June 10), pre-senescence (September 20) and late senescence (October 20), and 10 leaves of similar growth were randomly selected from each part of the tree canopy in different periods for measurement. Ten leaves color characteristics were measured at the tip and center of the leaf as well as at the base of the petiole to obtain an average. These measurements were repeated three times and the L*, a* and b* values were recorded, where L* indicates brightness on a 0–100-point scale (from black to white); a* represents colour on a green-red axis ranging from –120 to 120, anchored by green and red at the positive and negative ends, respectively; and b* represents the blue-yellow axis, which also ranged from –120 (blue) to 120 (yellow).

### Determination of chlorophyll, carotenoid and anthocyanin in leaves

Chlorophyll and carotenoid concentration were determined by direct ethanol extraction [21]. Fresh leaves were washed with distilled water and the veins were removed. The fresh leaves were then cut into fine strips about 1 mm wide, weighed to the nearest 0.1 g, and placed in a test tube to which 95% ethanol was added to make up a 10 mL volume. The test tube was sealed with plastic wrap and stored in the dark for 12–24 hours until the leaf strips turned completely white. The solution was then aspirated into a cuvette. The chlorophyll concentration was calculated by measuring the optical density values at 665, 649 and 470 nm with a spectrophotometer, using 95% ethanol as a blank control.


$${\text{C}}_{\text{a}}(\text{Chlorophyll}\;\text{a})=13.95D_{665}-6.88D_{649}$$

$${\text{C}}_{\text{b}}(\text{Chlorophyll}\;\text{b})=24.96D_{649}-7.32D_{665}$$

$$\text{C}={\text{C}}_{\text{a}}+{\text{C}}_{\text{b}}$$

$${\text{C}}_{\text{x}}{}_{\cdot \text{c}}(\text{carotenoid})=(1000D_{470}-2.05{\text{C}}_{\text{a}}-114.8{\text{C}}_{\text{b}})/245$$

$$\text{Chloroplast}\;\text{pigment}\;\text{concentration}\;\left(\text{mg}/\text{g}\right)=(\text{C}\times \text{VT}\times \text{n})/(\text{FW}\times 1000)$$

In the equations:

*D*_*470*_, *D*_*649*_ and *D*_*665*_ nm—Optical density of chlorophyll solution at wavelength 470,649 and 665;

Ca—Chlorophyll a concentration (mg·g^-1^)

Cb—Chlorophyll b concentration (mg·g^-1^)

C—concentration of chloroplast pigments (mg·g^-1^);

FW—fresh weight of the sample;

VT—total volume of extract;

n—dilution times.

The anthocyanin concentration was determined using the method of Li et al. [2015] [22]. Fresh leaves were cut and weighed to the nearest 0.1 g in a triangular flask containing 10 mL of 1 mol L^-1^ hydrochloric acid. This mixture was then placed in an oven at 32°C for 8 hours, after which it was centrifuged. The supernatant was then collected to determine its optical density at 530 nm and calculate the anthocyanin concentration.


$$\text{C}={\text{OD}}_{530}/(0.1\times \text{FW})$$

In the equation:

C—Anthocyanin concentration (U·g^-1^);

OD530—Absorbance value of sample solution;

FW—fresh weight of the sample.

### Measurement of biochemical indicators

Soluble protein concentration was determined by the Coomassie brilliant blue G-250 staining method [22]. Using the pure reagent as a blank, we determined the absorbance of a mixture of protein extract and Coomassie brilliant blue reagent at 595 nm with a spectrophotometer.

The soluble sugar concentration was determined by the anthrone colorimetric method [22]. The solution to be measured was mixed with anthrone sulphate and heated in boiling water for 10 minutes, after which it was cooled. The absorbance was measured at 620 nm.

The malondialdehyde (MDA) concentration was determined by the thiobarbituric acid method (TBA) [22]. A mixture of MDA extract and 0.6% TBA was heated in boiling water for 15 minutes, and centrifuged at 12,000 r/min at 4°C for 10 minutes. The absorbance of the supernatant was measured at 532, 600, and 450 nm.

Superoxide dismutase (SOD), peroxidase (POD) and catalase (CAT) activities were determined using the method of Wang et al. [2015] [21]. Take 0.5g of leaves in a pre-chilled mortar, add 1mL of pre-chilled phosphate buffer and grind into a slurry on an ice bath, adding buffer to make the final volume of 5mL. Centrifuge at 4000r/min for 10min, the supernatant is the enzyme extracts. Then, 3 mL of reaction mixture was prepared. The mixture contained 0.3 mL of 130 mmol L^-1^ methionine, 0.3 mL of 750 μmol L^-1^ NBT, 0.3 mL of 100 μmol/LEDTA-Na_2_, 0.3 mL of 20 μmol L^-1^ riboflavin, 1.5 mL of variable volume 50 mmol L^-1^ phosphate buffer, 0.25 mL of distilled water, and a 0.05 mL mixture of enzyme extracts. The reaction mixture was used to calculate SOD activity by measuring its absorbance at 560 nm.


$$\text{SOD}\;\text{total}\;\text{activity}\;(\text{U}/\text{g})=\left({\text{A}}_{\text{CK}}-{\text{A}}_{\text{E}}\right)\times {\text{V}}_{\text{T}}/(0.5\times {\text{A}}_{\text{CK}}\times \text{m}\times {\text{V}}_{\text{S}})$$

In the equation:

A_CK_ is the absorbance of the blank control; A_E_ is the absorbance of the sample; V_T_ is the total volume of the sample solution (mL); V_S_ is the amount of sample used in the measurement (mL); m is the fresh weight of the sample(g).

Take 0.5g of leaves cut up and put into a mortar. Add appropriate amount of phosphate buffer to grind into homogenate. Transfer all the homogenate into a centrifuge tube, centrifuge at 3000r/min for 10min, and transfer the supernatant into a 25mL volumetric flask. The precipitate was extracted twice more with 5mL phosphate buffer, and the volume was fixed to the scale. To calculate POD activity, 0.1 mL of enzyme extract was dissolved in 4.9 mL of solution containing 1.0 mL of 0.05 mol L^-1^ guaiacol, 1.0 mL of 2% H_2_O_2_, and 2.9 mL of 0.05 mol L^-1^ phosphate buffer. This enzyme solution was boiled for 5 minutes as a control, immediately transferred to a 37°C water bath for 15 minutes, and then quickly transferred to an ice bath, after which 2.0 mL of 20% trichloroacetic acid was added to terminate the reaction. The solution was centrifuged at 5,000 r min^-1^ for 10 minutes and diluted appropriately, and the absorbance was measured at 470 nm.


$$\text{POD}\;\text{activity}\left[\mu /\left(\text{g}\cdot \text{min}\right)\right]=\Delta {\text{A}}_{470}\cdot {\text{V}}_{\text{T}}/\left(\text{m}\cdot {\text{V}}_{\text{S}}\cdot 0.01\cdot \text{t}\right)$$

In the equation:

ΔA_470_ is the change in absorbance during the reaction time; m is the fresh weight of the sample(g); t is the reaction time(min); V_T_ is the total volume of extracted enzyme solution (mL); V_S_ is the volume of enzyme liquid used in the assay (mL).

Take 0.5g of leaf cut, add a small amount of phosphate buffer solution pH7.8, grind into a homogenate, transfer to a 25mL volumetric flask, rinse the mortar with the buffer, and transfer the rinse solution to the volumetric flask to fix the volume, centrifuge at 4000r/min for 15min, the supernatant is the enzyme solution. We added 2.5 mL of enzyme solution and 2.5 mL of the dead (control) enzyme solution to 2.5 mL of 0.1 mol L^-1^ H_2_O_2_. This mixture was kept in a constant-temperature water bath at 30°C for 10 minutes, after which we immediately added 2.5 mL of 10% H_2_SO_4_ and titrated the mixture with 0.1 mol L^-1^ of standard KMnO_4_ solution until it displayed a pink colour for at least 30 seconds. The CAT activity was then analysed.


$$\text{CAT}\;\text{activity}\;\left[\text{mg}/\left(\text{g}\cdot \text{min}\right)\right]\;=(\text{VT}/\text{VS)}\cdot (\text{A}-\text{B)}\cdot 1.7/\left(\text{m}\cdot \text{t}\right)$$

In the equation:

A is the control KMnO4 titration volume(mL); B is the titrated volume of KMnO4 after the enzyme reaction(mL); V_T_ is the total volume of extracted enzyme solution (mL); V_S_ is the volume of enzyme liquid used in the assay (mL); m is the fresh weight of the sample(g); t is the reaction time(min); 1.7 means 1 mL 0.1 mol/L KMnO_4_ is equivalent to 1.7 mg of H2O2.

### Metabolome analysis

First, 100 mg samples of freeze-dried powdered leaves were dissolved in 1 mL of 70% methanol solution (stored overnight at 4°C to allow complete dissolution), after which they were vortexed three times and centrifuged at 10,000 g for 10 minutes. The supernatant was filtered through a microporous membrane (0.22 μm) and collected into a sample bottle for liquid chromatography-mass spectrometry (LC-MS) analysis.

The LC-MS analysis was performed using ultra-high-performance liquid chromatography (UPLC) and a tandem mass spectrometry (MS/MS) system. The parameters for the linear ion trap and triple quadrupole of the QTRAP 4500 LC-MS/MS system (Sciex, Framingham, MA. USA) were as follows: electrospray ion (ESI) source temperature = 550°C, mass spectrum voltage = 5,500 V, curtain gas (CUR) pressure = 25 psi, and collision-activated dissociation (CD) parameter setting = high. In the triple quadrupole (QQQ), ion pairs were detected based on declustering potential (DP) and collision energy (CE). The data were processed with Analyst 1.6.1 software (SCIEX) and analysed using unsupervised principal component analysis (PCA) and supervised orthogonal partial least squares discriminant analysis (OPLS-DA). Public mass spectrometry databases (KNAPSACK, DB, METLIN, etc.) and an in-house metabolite database were referred to for qualitative and quantitative analysis of metabolites. We obtained variance influence on projection (VIP) values from the OPLS-DA analysis, and *P*-values from univariate t-tests, to identify significantly differentially expressed metabolites among groups [23]. The threshold values for significantly differential expression were VIP ≥ 1 and *P* < 0.05. Finally, the Kyoto Encyclopedia of Genes and Genomes (KEGG) database was used for pathway enrichment analysis of the differential metabolites.

## Results

### Variation in leaf colour parameters

The leaf colour parameters of *Q*. *mongolica* changed during senescence (Fig 1b). Leaf colour parameter L* increased at first and then tended to remain constant as leaves continued to senesce. The L* values of stage 2 and stage 3 were significantly higher than at stage 1, indicating that the brightness of senescing leaves was higher than that of mature green leaves. The leaf colour parameter a* also increased with leaf age. The a* values of stages 2 and 3 were significantly higher than at stage 1, indicating a colour change from green to red. Stage3 a* values were higher than at stage 2, but not significantly. The leaf colour parameter b* increased at first but then decreased slightly as leaves senesced. Stage 2 and 3 b* values were significantly higher than at stage 1, which reflected age-dependent leaf yellowing. Stage 3 b* values were lower than at stage 2, but not significantly. The slight decrease of b* in stage 3 was presumed to be caused by the drying of leaves at the early deciduous stage.

### Changes in leaf pigment concentration

The chlorophyll and carotenoid concentration of *Q*. *mongolica* leaves decreased over time. Chlorophyll a, chlorophyll b, total chlorophyll and carotenoids concentration all decreased significantly. The values for carotenoids/total chlorophyll were 0.15, 0.71 and 1.13 during stages 1–3, respectively, and the proportion of carotenoids increased over time. The anthocyanin concentration initially decreased and then increased significantly over time; in stage 2, it was 23.6% lower than in stage 1 but 10.7% higher than in stage 3 (Fig 1c).

### Analysis of relevant physiological indicators

**Fig 2:**
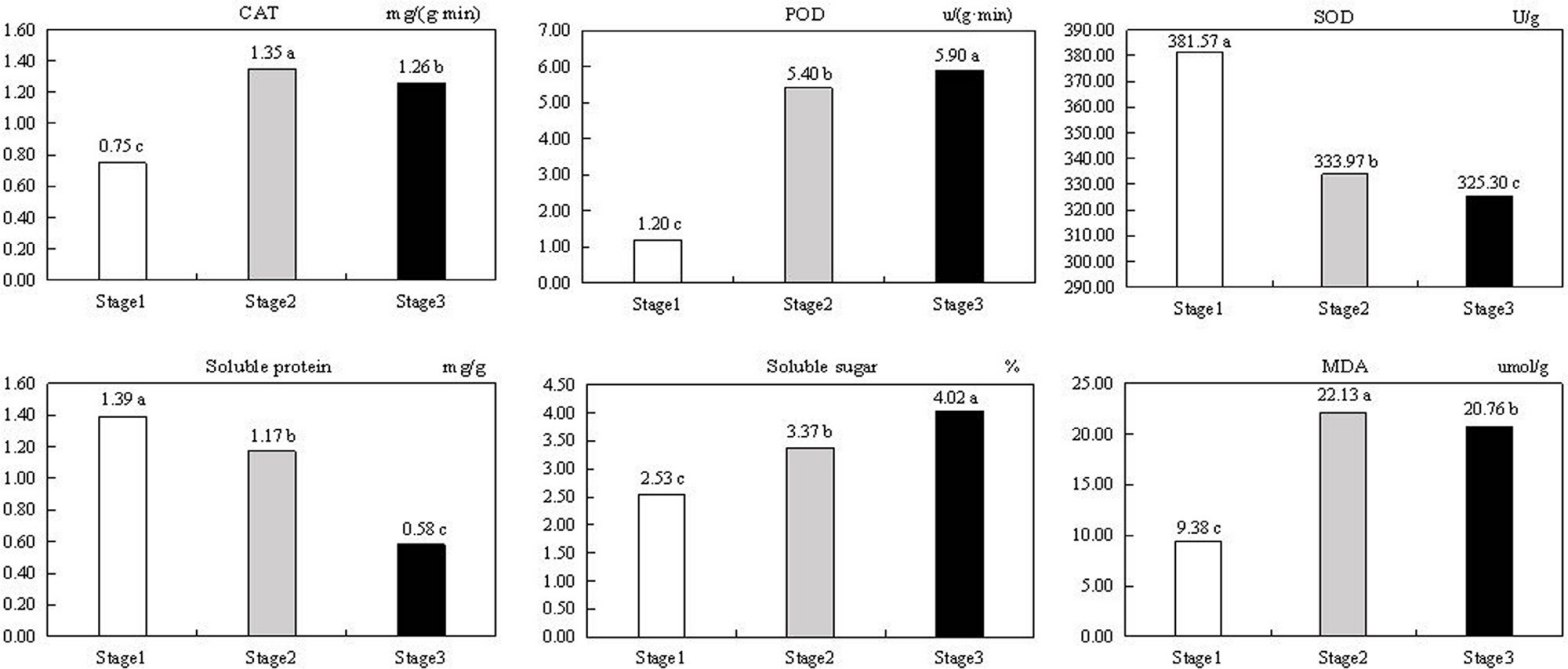
Osmoregulatory substances, protective enzymes, and MDA content.

We assessed the responses of *Q*. *mongolica* leaf cells to senescence-related oxidative and osmotic damage based on the activities of POD, SOD and CAT, and the leaf concentration of MDA, soluble proteins, and soluble sugars (Fig 2). Compared with stage 1, the soluble protein concentration decreased by 15.83% in stage 2 and 58.27% in stage 3. Similarly, the activity of SOD decreased by 12.47% in stage 2 and 17.74% in stage 3 relative to stage 1. By contrast, POD activity in stages 2 and 3 was 3.5 and 3.92 times greater than in stage 1, indicating that POD responded strongly to leaf senescence. Soluble sugars concentration were 33.2% and 58.90% higher in stages 2 and 3 relative to stage 1. Compared to stage 1, CAT activity increased by 80% in stage 2 and 68% in stage 3. Membrane lipid peroxidation occurs during leaf senescence, and MDA is one of its products, which is usually utilized to indicate the degree of membrane lipid peroxidation. MDA concentration initially increased and then decreased as senescence progressed. From these results, we deduce that *Q*. *mongolica* leaves experienced cell membrane damage during pre-senescence. And the MDA concentration decreased later, presumably because the increased activity of two antioxidant enzymes, CAT and POD, alleviated this damage.

### Multivariate statistical analysis

**Fig 3:**
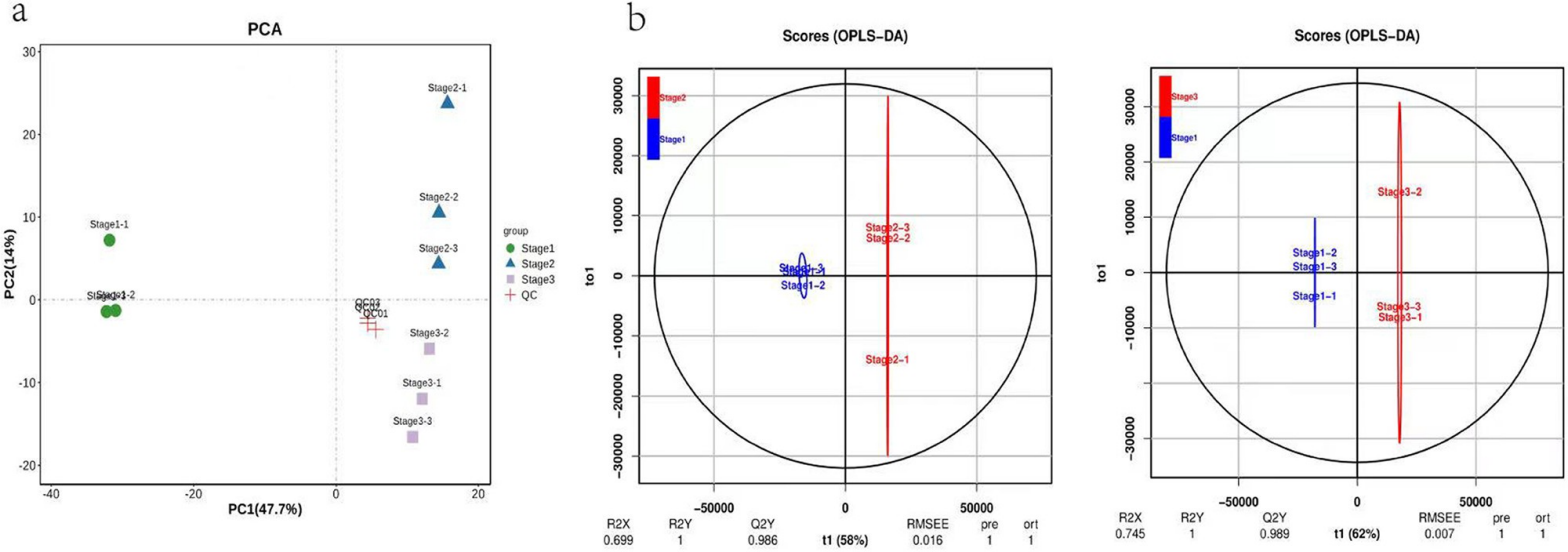
(a) PCA of leaf samples from different periods. (b) OPLS-DA of leaf samples obtained in three periods.

In the PCA biplot, the quality control samples were tightly clustered, indicating that the instrumental analysis was stable and reliable. The remaining three groups of samples could be clearly distinguished, indicating adequate experimental replication. PC1 and PC2 jointly explained > 61% of the variance in leaf biochemistry. PC1 clearly distinguished the experimental and control groups, and PC2 distinguished between the two experimental groups (Fig 3a). These results clearly demonstrated that *Q*. *mongolica* leaves differed significantly across the stages of senescence. The OPLS-DA score plot showed that data points in stage 1 were relatively concentrated, those in stages 2 and stage 3 were more scattered. There was substantial within-group variability in the rates of *Q*. *mongolica* leaf senescence (Fig 3b).

### Qualitative and quantitative analysis of metabolites

**Fig 4:**
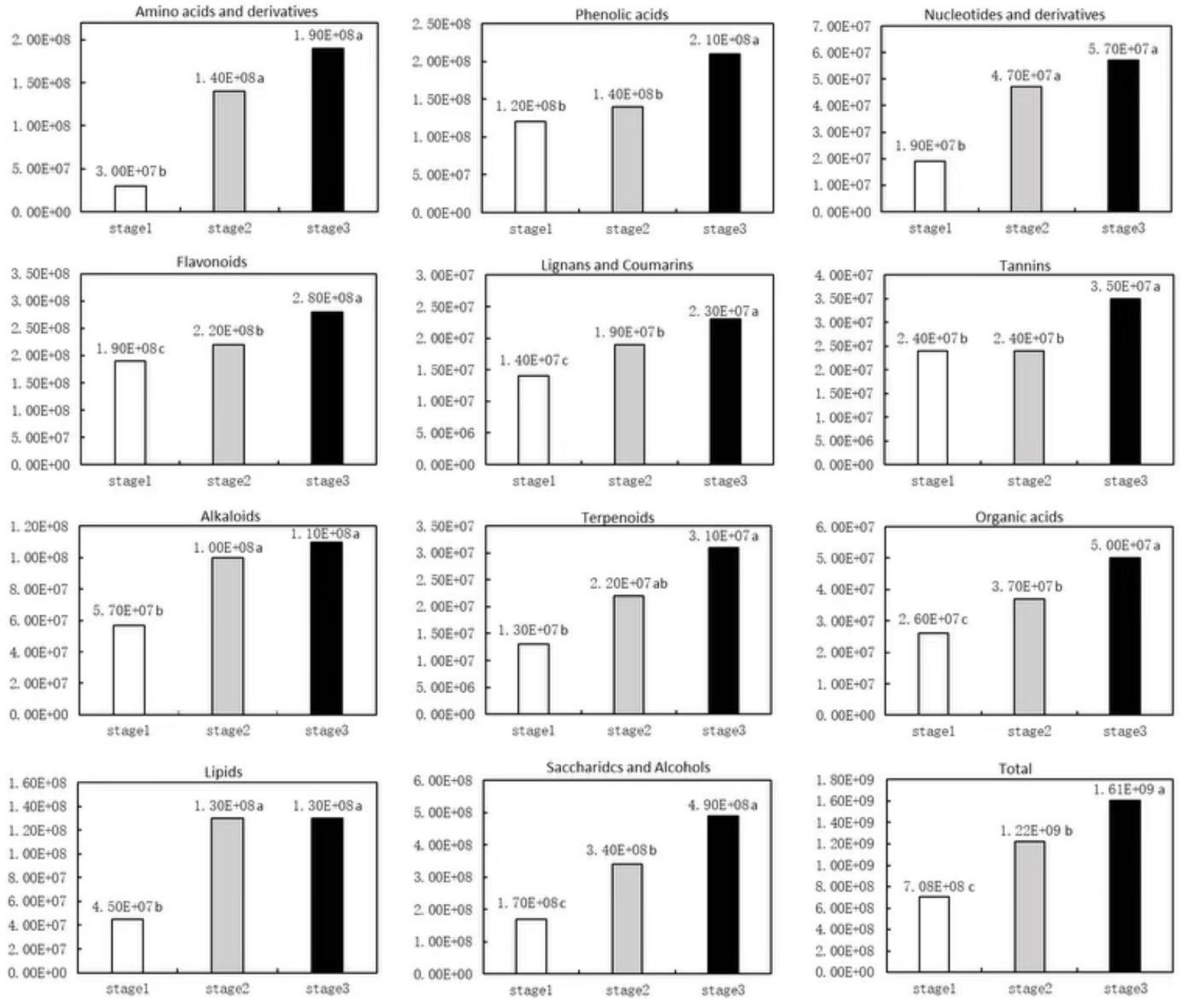
Metabolite contents during the three stages of senescence.

The widely targeted metabolomic analysis of *Q*. *mongolica* leaves from three time periods revealed 797 metabolites. These metabolites were classified into 11 classes: phenolic acids (n = 145), flavonoids (n = 118, including 3 chalcones, 9 dihydroflavonoids, 4 dihydroflavonols, 25 flavonoids, 61 flavonols, 4 flavonoid carbon glycosides, and 12 flavanols), lipids (n = 113), carbohydrates (n = 95), amino acids and their derivatives (n = 67), organic acids (n = 58), nucleotides and their derivatives (n = 54), lignans and coumarins (n = 50), alkaloids (n = 45), tannins (n = 33), and terpenoids (n = 19). The total numbers of metabolites were significantly higher in stages 2 and 3 than stage 1. The total metabolite content increased over the senescence period (Fig 4).

**Fig 5:**
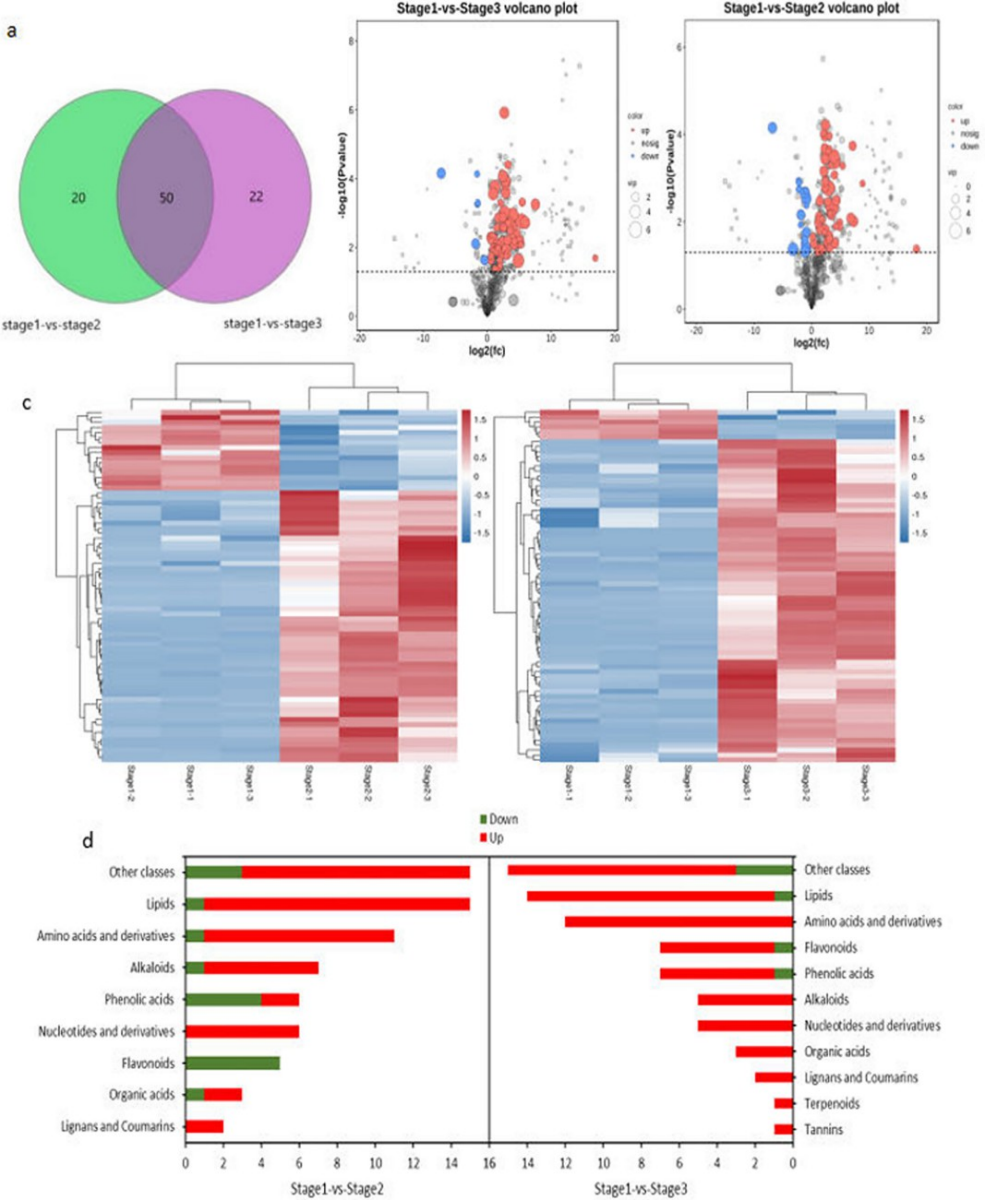
(a) Wayne diagram of differential metabolites. (b) Volcano plot of differential metabolites. (c) Heat map of differential metabolites exhibiting up- and down-regulated expression. (d) Number of functional classes and differential metabolites.

Seventy metabolites distinguished stages 1 and 2. Of these metabolites, 54 had higher values in stage 2 than stage 1, while 16 had lower values in stage 2. Lipid metabolites and carbohydrate compounds constituted the largest metabolite groups in stage 2, and together accounted for 21.43% of all metabolites. In total, 66 differential metabolites were upregulated, and 6 were downregulated, in stage 3 versus stage 1. The dominant metabolite groups in stage 3 were carbohydrates, lipids, and amino acids and their derivatives, which accounted for 20.83%, 19.44%, and 16.67% of all metabolites, respectively. Differential metabolites common to stages 2 and 3, and those that occurred in only one stage, were visualised using Venn diagrams. The two stages had 50 differential metabolites in common, while 22 were unique to stage 2 and 20 to stage 3 (Fig 5a).

A volcano plot helped us to identify differential metabolites more accurately (Fig 5b), while a heat map showed group differences in the accumulation of differential metabolites (Fig 5c). The results suggest that leaf senescence promotes upregulation of a number of metabolites. Differential metabolites were classified into 11 classes based on their molecular structure and function (Fig 5d). Notably, no tannin or terpene metabolites were significantly upregulated during stage 2 relative to stage 1, although one tannin and one terpene metabolite were significantly increased during stage 3 (Fig 5d). We identified the top 20 upregulated and downregulated differential metabolites in comparisons of stages 1 and 2, and stages 1 and 3 (Table 1).

**Table 1:** The top 20 upregulated and downregulated differential metabolites.

	Class	Compounds	log2_FC
**Stage1 vs Stage3**	Phenolic acids	2,3,4-Trihydroxybenzoic acid	18.2157124
	Alkaloids	N-Oleoylethanolamine	8.846719257
	Alkaloids	DL-2-Aminoadipic acid	7.306316615
	Nucleotides and derivatives	2’-O-Methyladenosine	7.11422113
	Organic acids	2-Hydroxyhexadecanoic acid	6.811188576
	Lipids	9,12,13-TriHOME;9(S),12(S),13(S)-Trihydroxy-10(E)-octadecenoic acid	5.398846008
	Lignans and Coumarins	Pinoresinol dimethy ether	4.881901084
	Lignans and Coumarins	Dimethylmatairesinol	4.858492162
	Amino acids and derivatives	L-Tryptophan	4.273725363
	Amino acids and derivatives	N-Glycyl-L-leucine	4.21506039
	Alkaloids	3-Indoleacrylic acid	4.040974617
	Amino acids and derivatives	L-Norleucine	3.98116765
	Amino acids and derivatives	3-Hydroxy-3-methylpentane-1,5-dioic acid	3.965028907
	Alkaloids	3-amino-2-naphthoic acid	3.832906436
	Amino acids and derivatives	L-Proline	3.557883467
	Amino acids and derivatives	L-Histidine	3.161119999
	Lipids	LysoPC 18:3	3.402113966
	Lipids	LysoPC 16:0(2n isomer)	3.320907117
	Lipids	LysoPC 17:0(2n isomer)	3.219598229
	Lipids	LysoPC 17:0	3.219598229
	Lipids	2-Dodecenedioic acid	-6.815556272
	Phenolic acids	Neochlorogenic acid (5-O-Caffeoylquinic acid)	-3.228652871
	Others	Glucose-1-phosphate	-2.255440616
	Others	D-Glucose 6-phosphate	-2.209552578
	Others	D-Fructose 6-Phosphate	-2.151486395
	Phenolic acids	1-O-Feruloylquinic acid	-1.907307371
	Phenolic acids	Homovanilloylquinic acid	-1.872714257
	Phenolic acids	4,6-(S)-Hexahydroxydiphenoyl-β-D-glucose	-1.224283687
	Flavonoids	Epicatechin	-1.181303382
	Flavonoids	Kaempferol-3-O-(6’’-galloyl)glucoside	-1.164166285
	Flavonoids	Kaempferol-3-O-(2’’-acetyl)glucoside	-1.080016981
	Flavonoids	Quercetin-3-O-(6’’-O-galloyl)galactoside	-0.942794349
	Amino acids and derivatives	Methyl 3-aminopropanoate	-0.941918232
	Flavonoids	Catechin	-0.933652134
	Alkaloids	Choline	-0.908994351
	Organic acids	2-Aminoisobutyric acid	-0.882131479
	Lipids	2,3,4-Trihydroxybenzoic acid	16.90716017
	Alkaloids	DL-2-Aminoadipic acid	7.532794305
	Amino acids and derivatives	L-Valine	5.927777912
	Alkaloids	2-Hydroxyhexadecanoic acid	5.472080338
	Amino acids and derivatives	L-Isoleucine	5.34281071
	Amino acids and derivatives	2’-O-Methyladenosine	5.208056804
	Lignans and Coumarins	Dimethylmatairesinol	5.033978754
	Amino acids and derivatives	L-Norleucine	4.978471978
	Amino acids and derivatives	L-Leucine	4.861618045
	Nucleotides and derivatives	Pinoresinol dimethy ether	4.722897341
	Amino acids and derivatives	N-Glycyl-L-leucine	4.23892602
	Amino acids and derivatives	L-Tryptophan	4.1655589
	Amino acids and derivatives	L-Histidine	4.153320295
	Alkaloids	3-Indoleacrylic acid	3.986271499
	Amino acids and derivatives	L-Proline	3.816307069
	Lignans and Coumarins	3-amino-2-naphthoic acid	3.796776305
	Amino acids and derivatives	3-Hydroxy-3-methylpentane-1,5-dioic acid	3.551317391
	Lipids	LysoPC 18:3	3.538336353
	Organic acids	LysoPE 18:3(2n isomer)	3.26976807
	Lipids	L-Homoserine	3.238884372
	Lipids	2-Dodecenedioic acid	-7.211844399
	Phenolic acids	Homovanilloylquinic acid	-1.829081471
	Others	D-Fructose 6-Phosphate	-1.590155966
	Others	D-Glucose 6-phosphate	-1.548982589
	Others	Glucose-1-phosphate	-1.4837736
	Flavonoids	Epicatechin	-0.453064854

**Fig 6:**
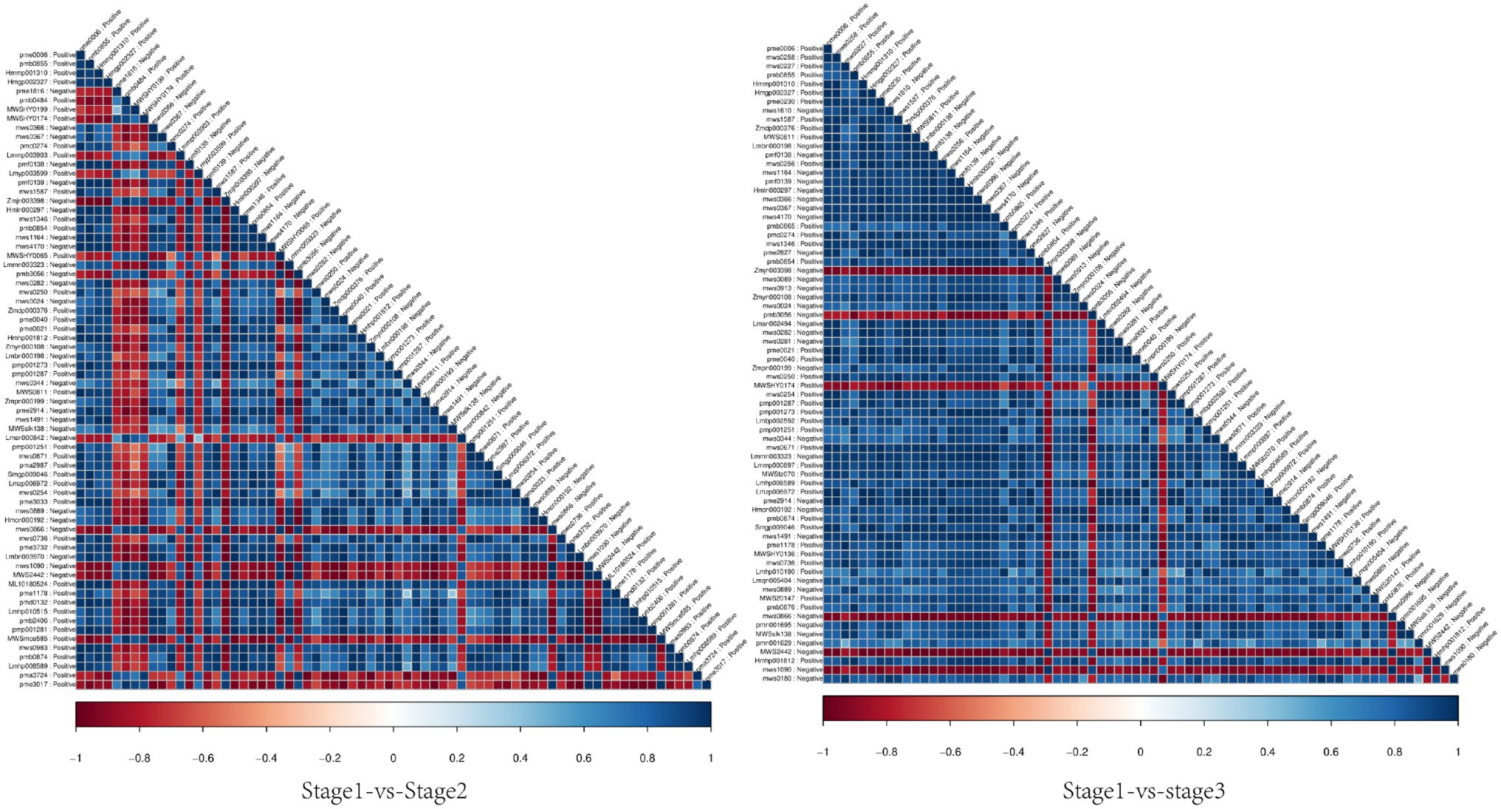
Heat map showing correlations among differential metabolites across groups. Note: Correlations between individual metabolites were analysed by Pearson correlation. The correlation coefficients take values between 1 and -1.

We used Pearson correlation to investigate the consistency and direction of changes in the relationships between all possible pairs of metabolites in each group (Fig 6). In the stage 1 versus stage 2 comparison, there were 1,179 positive and 1,247 negative correlations among 70 differential metabolites. In the stage 1 versus stage 3 comparison, there were 1,760 positive and 357 negative correlations among 72 differential metabolites.

### Metabolic pathway analysis of differential metabolites

To gain further insight into the differential metabolites in metabolic pathways, we subjected the differential metabolites to KEGG pathway annotation and enrichment analysis. As seen in Table 2, the differential metabolites in the stage 1 versus stage 2 and stage 1 versus 3 comparisons were principally enriched in amino acid metabolism, lipid metabolism, and the biosynthesis of secondary metabolites. The differential metabolites in the stage 1 versus 2 comparison mapped to 53 metabolic pathways; there were 13 significantly enriched pathways, including glycine, serine and threonine metabolism, starch sucrose metabolism, insulin resistance, aminoacyl-tRNA biosynthesis, biosynthesis of antibiotics, galactose metabolism, biosynthesis of unsaturated fatty acid, ascorbate and aldarate metabolism, and indole alkaloid biosynthesis. Biosynthesis of amino acids, ABC transporter, and thioglucoside pathways were also enriched.

**Table 2:** KEGG pathway annotations for the differential metabolites in different growth periods.

Category	Number of DMs	
	Stage 1 vs. stage 2	Stage 1 vs. stage 3
Amino acid metabolism	31	37
Lipid metabolism	22	19
Biosynthesis of secondary metabolites	14	18
Energy metabolism	3	3
Carbohydrate metabolism	11	12
Membrane transport	7	9
Metabolism of cofactors and vitamins	1	2
Metabolism of other amino acids	3	5
Metabolism of terpenoids and polyketides	1	2
Nucleotide metabolism	12	11
Translation	5	8

Differential metabolites in the stage 1 versus 3 comparison mapped to 59 metabolic pathways; there were 17 significantly enriched pathways, including biosynthesis of glucosinolate, aminoacyl-tRNA, antibiotics, amino acids, unsaturated fatty acids, valine, leucine, isoleucine, tropane, piperidine, and pyridine and indole alkaloids. Other enriched pathways included cyanoamino acid metabolism, 2-oxocarboxylic acid metabolism, ABC transporter, starch and sucrose metabolism, valine, leucine and isoleucine degradation, insulin resistance pathway, galactose metabolism, and ascorbate and aldarate metabolism.

**Fig 7:**
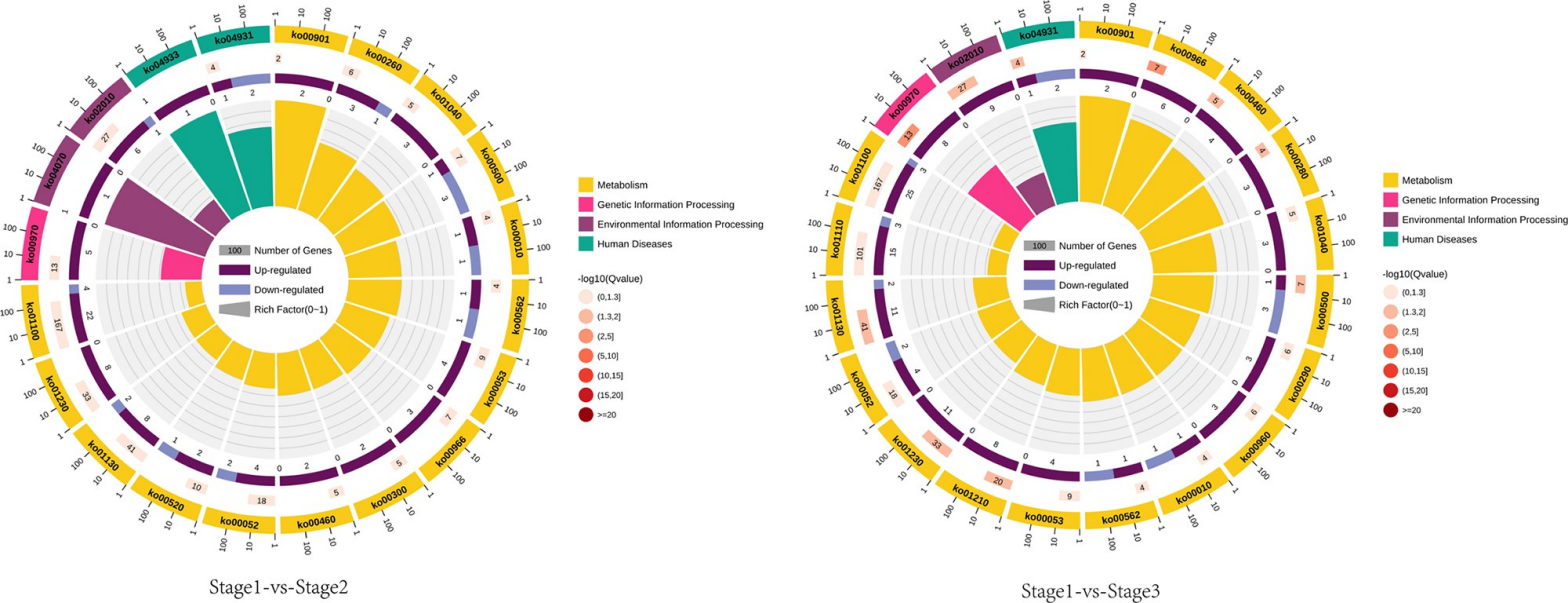
Results of KEGG enrichment analysis. The first circle shows the top 20 enriched pathways. The scale denotes the number of differential metabolites. Different colours represent different classes. The second circle shows the number of pathways in the “differential metabolite background”, and the Q values. Higher numbers of differential metabolites are represented by longer bars, and smaller Q values by a redder colour. The third circle shows the upregulated and downregulated differential metabolite ratios. Dark and light purple represent upregulation and downregulation, respectively. Values are shown below the bars. The fourth circle shows the enrichment values (number of differential metabolites in a given pathway divided by the total number of metabolites in that pathway). Each cell in the background gridlines represents 0.1.

Regarding the differential metabolites in stages 2 and 3, 12 pathways were significantly enriched, including glucosinolate biosynthesis, aminoacyl-tRNA biosynthesis, biosynthesis of antibiotics and amino acids, ABC transporter, starch and sucrose metabolism, insulin resistance pathway, indole alkaloid biosynthesis, biosynthesis of unsaturated fatty acid, galactose metabolism, and ascorbate and aldarate metabolism. The differential metabolites of stage 2 were also significantly enriched in the glycine, serine and threonine metabolic pathways, while the differential metabolites of stage 3 were also significantly enriched in the cyanoamino acid metabolism, 2-oxocarboxylic acid metabolism, valine, leucine and isoleucine degradation, valine, leucine and isoleucine biosynthesis, tropane, piperidine and pyridine alkaloid biosynthesis and secondary metabolite biosynthetic metabolic pathways. The top 20 enriched metabolic pathways are illustrated in Fig 7, which shows the relationships between differential metabolites and metabolic pathways (Fig 7).

### Analysis of changes in key metabolites in response to aging

**Fig 8:**
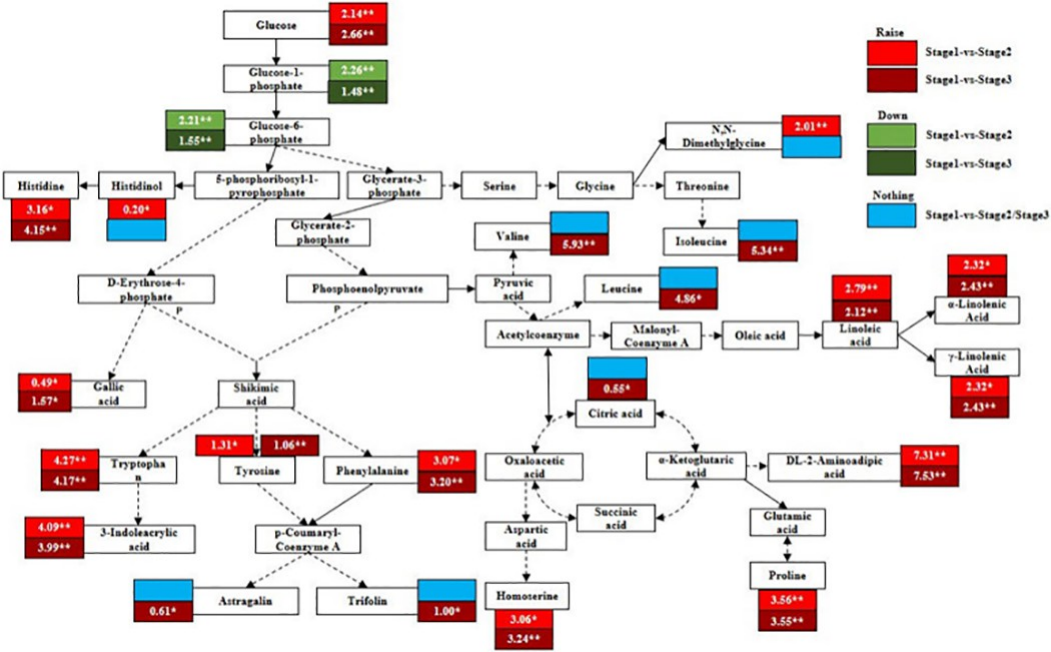
Changes in differential metabolites with ageing. Note: Boxes filled in white represent differential metabolites, numbers in boxes represent fold changes, light red and dark red boxes represent differential metabolites upregulated in stages 2 and 3, respectively, light green and dark green boxes represent differential metabolites downregulated in stages 2 and 3, respectively, and blue boxes represent differential metabolites absent in stages 2 and 3 *P<0.05; **P<0.01.

To analyse changes in key metabolites in response to ageing, we used the data for differential metabolites showing pathway enrichment in stages 2 and 3 to create a simple metabolic map (Fig 8). This figure shows important metabolic pathways including starch and sucrose metabolism, the citrate cycle (TCA cycle), unsaturated fatty acid metabolism, biosynthesis of antibiotics, glycine, serine and threonine metabolism, arginine and proline metabolism, phenyl propane biosynthesis, and flavonoid biosynthesis. Fig 8 shows changes in metabolites during stages 2 and 3 compared to the mature (green leaf) stage, i.e. stage 1. Overall, leaf senescence had relatively large effects on the type and content of metabolites during amino acid metabolism, unsaturated fatty acid metabolism and secondary metabolite synthesis; it had smaller effects on metabolite production during the TCA cycle and sugar metabolism. Because the metabolite content values are large, they are illustrated as fold changes in Fig 8.

In amino acid metabolism, the contents of proline and tryptophan in stages 2 and 3 increased significantly (*P* < 0.01) compared with stage 1. Tyrosine, histidine, homoserine and phenylalanine increased significantly in stages 2 (*P* < 0.05) and 3 (*P* < 0.01). However, N, N-dimethylglycine only showed a significant increase in stage 2 (*P* < 0.01), while valine, leucine and isoleucine only increased significantly in stage 3 (*P* < 0.01).

In unsaturated fatty acid metabolism, 1,linoleic acid, γ-linolenic acid and α-linolenic acid were highly significantly upregulated in stages 2 and 3 (*P* < 0.01) compared with stage 1. In secondary metabolite biosynthesis, the levels of DL-2-aminoadipic acid and 3-indoleacrylic acid were significantly upregulated (*P* < 0.01) in stages 2 and 3 compared with stage 1. Notable upregulation was observed for DL-2-aminoadipic acid in stages 2 (7.31) and 3 (7.53), and for 3-indoleacrylic acid in stages 2 (4.0) and 3 (3.99). Gallic acid was significantly upregulated (*P* < 0.05) in stages 2 and 3, while histidinol was significantly upregulated in stage 2, and trifolin and astragalin were significantly upregulated in stage 3 (*P* < 0.05).

In the TCA cycle, citric acid was significantly upregulated (*P* < 0.05) in stage 3. In glucose metabolism, glucose was significantly upregulated while fructose-6-phosphate, glucose-6-phosphate and glucose-1-phosphate were significantly downregulated in stages 2 and 3 (all *P* < 0.01). In addition, 2,3,4-trihydroxybenzoic acid, N-oleoylethanolamine, 2-hydroxyhexadecanoic acid, 2-methoxyadenosine, pinoresinol dimethyl ether, dimethylmatairesinol, N-glycyl-L-leucine, 3-hydroxy-3-methylpentane-1,5-dioic acid and 3-amino-2-naphthoic acid were significantly upregulated in stage 2 (*P* < 0.01), although they lacked metabolic pathway annotations. Upregulation was particularly marked for 2,3,4-trihydroxybenzoic acid and oleoyl monoethanolamine, which had fold changes of 18.22 and 8.85, respectively. In stage 3, 2,3,4-trihydroxybenzoic acid (*P* < 0.05) and 2-hydroxyhexadecanoic acid, 2’-O-methyladenosine, dimethylmatairesinol, L-norleucine, pinoresinol dimethyl ether, N-glycyl-L-leucine, 3-amino-2-naphthoic acid and 3-hydroxy-3-methylpentane-1,5-dioic acid (*P* < 0.01) all were all significantly upregulated. The upregulation was especially marked in 2,3,4-trihydroxybenzoic acid and 2-hydroxyhexadecanoic acid, which showed fold changes of 16.91 and 5.47, respectively. Although most of these metabolites were secondary metabolites, lipids, organic acids, amino acids and their derivatives were also represented, as well as nucleotides and their derivatives. These compounds are presumed to play important roles in the resistance of *Q*. *mongolica* leaves to senescence.

## Discussion

Plant leaf senescence leads to changes in leaf colour, physiology, and biochemistry, including the accumulation of osmoprotectants, synthesis of antioxidants, and changes in metabolic pathways [10, 20, 24]. In this study, we found significant differences in the metabolite contents of *Q*. *mongolica* leaves during leaf senescence stages 2 and 3 relative to stage 1 (mature stage). Further screening revealed a large number of differential metabolites in stages 2 and 3, most of which were amino acids and their derivatives or lipids. The number of differential metabolites was higher in stage 3 than stage 2, which indicated more intense metabolite changes during late senescence.

Differential metabolites in stages 2 and 3 were enriched in amino acids, lipid metabolism and the biosynthesis of secondary metabolites, indicating that these metabolites play important roles in *Q*. *mongolica* leaf senescence. *Q*. *mongolica* natural aging will experience low temperature, some studies have shown that low temperature stress will induce a large accumulation of reactive oxygen species (ROS) in plants, resulting in a series of oxidative damage such as membrane lipid peroxidation, proteins and nucleotides, thereby inducing cell death [19, 25]. It also leads to the synthesis of large amounts of protective substances such as soluble sugars (including sucrose, already sugar, cottonseed sugar, glucose, fructose and alginate) and proline, which can regulate cellular osmotic potential and stabilize cell membranes and protoplasts [26–29]. These processes produce nutrients that are transported to developing organs like fruits and seeds [30]. In summary, during leaf senescence, large quantities of accumulated metabolites originate from the hydrolysis of macromolecules and synthesis of primary and secondary metabolites. These compounds help plants to maintain a stable internal environment and contribute to reproductive development.

### Physiological response of *Quercus mongolica* leaves during senescence

Quantifying leaf color values by measuring leaf color parameters can reduce the error of subjective evaluation on the assessment of plant ornamental value [31]. In this study, compared to the mature stage, the L *, a *, and b * during the aging stage significantly increased. The significant increase in L * is due to the fact that plants change from green to yellow during aging, and yellow has a higher brightness and saturation than green. a* is a negative value during maturity, and a significant increase during senescence indicates that the leaves are green during maturity, while browning occurs during senescence. A significant increase in b * indicates yellowing of the leaves, which is consistent with human visual perception.

Changes in plant leaf color are directly due to changes in the spatial and temporal distribution of chlorophyll, carotenoid and anthocyanin concentration, while changes in pigment type and concentration are influenced by a combination of intrinsic genetic factors and external environmental factors [32–34]. In the present study, chlorophyll a, chlorophyll b, total chlorophyll and carotenoids were significantly decreased during senescence compared with maturity, which is consistent with the results of Wang’s and Gong’s study [35, 36]. Chlorophyll decomposition was accelerated and chlorophyll was significantly reduced in senescing leaves due to lower temperature and reduced light in autumn. In contrast, low temperature induced accelerated synthesis of anthocyanin [20], and anthocyanin increased significantly in the late senescence period.

SOD, POD and CAT are important antioxidant enzymes in plants with roles in membrane protein inhibition and peroxidation; their activities are closely related to plant stress resistance [37, 38]. SOD can convert harmful superoxide radicals into hydrogen peroxide (H_2_O_2_) and oxygen (O_2_). Hydrogen peroxide can be further reduced to water and O_2_ by POD and CAT, which can reduce the toxic effects of reactive oxygen species on the plant [39]. In this study, we found that SOD activity decreased in ageing *Q*. *mongolica* leaves, while POD activity increased, and CAT activity and MDA concentration first increased and then decreased. The decrease in SOD activity resulted in significant accumulation of superoxide radicals, which led to unsaturated lipid peroxidation in the cell membrane. MDA is a peroxidation product that can strongly react with various intracellular components and causes serious damage to enzymes and cell membranes. MDA-damaged membranes have reduced resistance and fluidity, and ultimately lose their physiological and structural integrity [40]. In this study, the significant increase in POD and CAT activities effectively inhibited membrane lipid peroxidation, which in turn led to a significant decrease in MDA concentration at the late stage of senescence. The decrease in MDA concentration can help alleviate the damage to cell membrane structure and function, delay leaf senescence, and allow more nutrients to flow to seeds and fruits. Therefore, it is hypothesized that POD and CAT are the main factors underlying the resistance of *Q*. *mongolica* leaves to senescence.

Soluble sugars and proteins are involved in osmoregulation in cells [41]. Soluble sugars, including monosaccharides and oligosaccharides, are the main product of photosynthesis. We observed that soluble sugars increased, while soluble proteins decreased, during senescence, presumably because protein hydrolysis caused a decrease in soluble protein concentration during ageing, while soluble sugar concentration increased to maintain a stable intracellular environment and provide energy for leaf metabolism. This is consistent with Mutsumi Watanabe’s research on Arabidopsis [42].

### Changes in metabolites during leaf senescence in *Quercus Mongolica*

Amino acids and proteins are nitrogen-containing organic compounds that constitute the main products of nitrogen and sulphur metabolism under environmental stress [43]. Protein degradation is a fundamental feature of leaf senescence [44]. As precursors of many secondary metabolites, these biomolecules are at the core of glycolysis, the pentose phosphate pathway, and the tricarboxylic acid cycle [45, 46]. In this study, amino acids and derivatives such as histidine, proline, tryptophan, phenylalanine, and tyrosine showed significant accumulation in senescence stages 2 and 3, and we surmise that the accumulation of these amino acids represents a strategy to maintain osmotic balance during leaf senescence [47].

Proline is an important component of protein formation in cell walls. This amino acid is responsive to increased levels of reactive oxygen species and helps to stabilise subcellular structures, eliminate reactive oxygen species, and regulate redox balance. Proline can also act as an osmotic agent that can regulate the internal environment of cells [48, 49]. Tryptophan is a precursor to many secondary metabolites, such as growth hormones, thioglucosides and alkaloids [50], which play important roles in plant growth, development, and antioxidant activity. Tyrosine, tryptophan and phenylalanine are aromatic amino acids in the shikimic acid pathway [51]. Phenylalanine is involved in the biosynthesis of many phytochemicals and antioxidants in the phenylpropanoid pathway [52], and as a polyphenol it protects tissues from oxidative damage by preventing free radical activity. Phenylalanine is also a precursor to the synthesis of secondary metabolites such as flavonoids. Histidine is an anti-inflammatory and antioxidant factor capable of regulating the synthesis of a wide range of amino acids [53, 54]. In the present study, the content of N,N-dimethylglycine increased significantly only at senescence stage 2, and the content of leucine, isoleucine and valine increased significantly only at stage 3, suggesting that *Q*. *mongolica* leaves adopt different resistance strategies at different stages of senescence. N,N-dimethylglycine is a chemical complex that appears in the choline to glycine metabolism pathway. The accumulation of N,N-dimethylglycine facilitates the synthesis of glycine. It has been shown that the addition of N,N-dimethylglycine salts to the feed of sows during lactation can treat immune dysfunction in piglets [55]. Valine, leucine and isoleucine are derived from pyruvate [56], which is a by-product of glycolysis, and it is possible that the high energy demands of plant leaves lead to the accumulation of these metabolites. It is noteworthy that, in this study, the contents of amino acids and their derivatives increased significantly in *Q*. *mongolica* leaves during senescence. This result was inconsistent with the findings of the rice leaf senescence study [57]. These inconsistencies may be caused by interspecific differences in metabolism, or by the need for intracellular environmental stability.

Lipids are important physiologically active substances and signalling molecules that are involved in cell metabolism and energy storage. They also stabilise the cytoskeleton [10]. The disruption of membrane structure during plant senescence induces an increase in fatty acid desaturase activity, which results in an increase in unsaturated fatty acid content, decrease in membrane lipid saturation, and increase in membrane fluidity, all of which contribute to the stability of the plant membrane system. In this study, lysophosphatidylcholine, lysophosphatidylethanolamine, γ-linolenic acid, α-linolenic acid and linoleic acid were significantly upregulated during senescence stages 2 and 3. Since linoleic acid is an unsaturated fatty acid with reducing properties, it is presumed to stabilise the structure and permeability of membrane lipids. Lysophospholipids are involved in different biological processes as intermediates of lipid metabolism and important signalling molecules [58, 59]. Lysophosphatidylethanolamine may retard chlorophyll breakdown and reduce electrolyte leakage by reducing the release of CO_2_ and ethylene [60]. Significant changes in lipid metabolites may be a response to senescence.

The content of organic acids (especially those in the TCA cycle) reflects the growth and metabolic activity of the plant. The TCA cycle links sugar and amino acid metabolism, provides energy to the plant, and mitigates plant stress. In this study, citric acid, which is one of the intermediate products of the TCA cycle, was 0.55 times higher in senescence stage 3 than stage 1. This result suggests that the TCA cycle may be enhanced in leaves during late senescence, and that it may produce provide additional energy to supplement energy consumption during senescence.

Secondary metabolites play important, and sometimes essential, roles in many aspects of plant life [61]. The wide variety of secondary metabolites in plants is a result of the long-term evolutionary adaptation of plants to their environments. Moreover, secondary metabolites participate in complex biological functions. Phenolic acids are widely distributed secondary metabolites in plant species. They contain several phenolic hydroxyl groups on their benzene rings that promote antioxidant and anti-free radical activities. They can therefore help plants to slow down the accumulation of free radicals caused by biotic and abiotic stresses.

We found that 2,3,4-trihydroxybenzoic acid was the main antioxidant in *Q*. *mongolica* leaves, and was strongly upregulated in senescence stages 2 and 3. Gentisic acid has antioxidant properties, and can reduce the severity of rheumatoid arthritis and prevent cancer [62, 63]. Gallic acid is a natural polyphenolic compound with antibacterial, anti-inflammatory, antioxidant, antiviral, antitumor and immunomodulatory properties [64].

Alkaloids are a large class of heterocyclic nitrogenous organic compounds of amino acid origin present in about 20% of plant species, often in the free state, as salts or nitrogen oxides that are widely distributed in plant vesicles. Alkaloids are often the main active ingredients of medicinal plants, and have anti-inflammatory, analgesic, and antiplasmodial properties, as well as insecticidal and hepatoprotective functions [65–67]. DL-2-aminoadipic acid belongs to the alkaloid group and is involved in the biosynthetic pathway of penicillin and cephalosporin. Histidinol is an upstream metabolite of histidine with anti-inflammatory and antioxidant properties. Therefore, a significant increase of alkaloids may protect leaves against the age-related production of reactive oxygen species.

Flavonoids are an important class of secondary metabolites in plants. Moreover, they are important polyphenolic compounds with antioxidant and free radical scavenging properties, are heavily involved in plant metabolism, and contribute to stress and disease resistance, growth, and development [68, 69]. When plants are in adverse environments, oxygen radicals accumulate in their cells due to metabolic disorders. Under these conditions, plants will increase their free radical scavenging and antioxidant activities by synthesising a large number of flavonoid substances.

In this study, epicatechin, kaempferol-3-O-(6’’-gallate)glucoside, kaempferol-3-O-(2’’-O-acetyl)glucoside, quercetin-3-O-(6’’-O-gallate)galactoside, and catechin were significantly downregulated during senescence stage 2. There were no significantly upregulated individual flavonoid metabolites, but total flavonoid metabolites were significantly upregulated (Fig 4). During stage 3, kaempferol-3,7-O-diglucoside, kaempferol-7-O-glucoside, kaempferol-3-O-galactoside (trifolin), kaempferol-3-O-glucoside (astragalin), gallocatechin-(4α→8)-gallocatechin, and l uteolin-3’-O-glucoside were also significantly upregulated. We hypothesise that these results reflect the adoption of different coping strategies by *Q*. *mongolica* leaves during different stages of senescence.

## Conclusion

We analysed physiological responses and metabolite compositions in *Quercus mongolica* leaves during three growth stages (between maturity and senescence). *Quercus mongolica* leaves induced significant accumulation of soluble sugars, significant decreases in soluble protein concentration, and significant reductions in chlorophyll concentration during senescence. POD and CAT were the main antioxidant enzymes that promoted leaf resistance to senescence. The amino acid metabolism, lipid metabolism and the biosynthesis of secondary metabolites are the main metabolic pathway during senescence. However, the contents of oleoyl monoethanolamine, histidinol, N,N-dimethylglycine, trifolin, astragalin, valine, isoleucine, leucine, citric acid, histidine, homoserine, tryptophan, tyrosine, phenylalanine, proline, n-leucine, N-glycyl-L-leucine, linoleic acid, linolenic acid, gallic acid, 3-indoleacrylic acid, 3-amino-2-naphthoic acid, 3-hydroxy-3-methylpentane-1,5-dioic acid, 2,3,4-trihydroxybenzoic acid, trifolin, astragalin, DL-2-aminoadipic acid, pinoresinol dimethyl ether, dimethyl resorcinol and lysophosphatidylcholine are the main metbolites. These chemical species constitute the key metabolites produced in *Q*. *mongolica* leaves in response to senescence.

## Supporting information

S1 Fig Integral correction diagram for quantitative metabolite analysis (negative). (PNG)

S2 Fig Integral correction diagram for quantitative metabolite analysis (positive). (PNG)

S3 Fig MRM detection metabolite multi-peak map (negative). (PDF)

S4 Fig MRM detection metabolite multi-peak map (positive). (PDF)

S5 Fig TIC overlap diagram of QC sample (negative). (PDF)

S6 Fig TIC overlap diagram of QC sample (positive). (PDF)

S7 Fig TIC diagram of QC sample (negative). (PDF)

S8 Fig TIC diagram of QC sample (positive). (PDF)

S1 Table Analysis of the results. (XLSX)
