# Supplementary figures and images for: Integrated physiological and metabolomic analyses reveal changes during the natural senescence of Quercus mongolica leaves
Source: PLoS One. 2023 Aug 23;18(8):e0289272. doi: 10.1371/journal.pone.0289272 (PMC10446833; doi:10.1371/journal.pone.0289272)

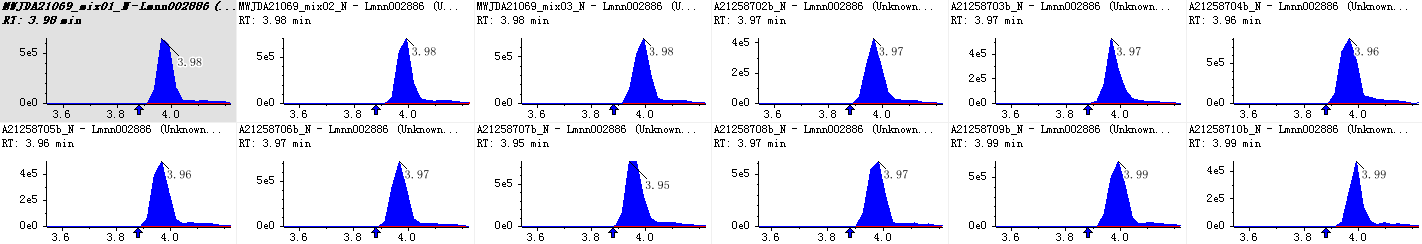

Supplement: S1 Fig — (PNG) [file pone.0289272.s001.png]

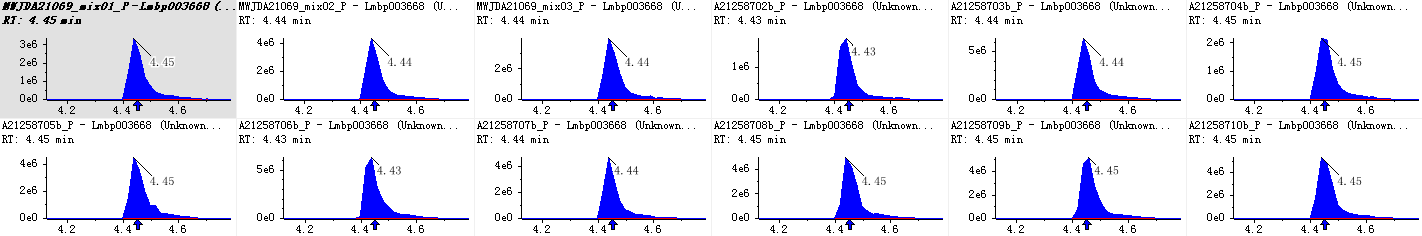

Supplement: S2 Fig — (PNG) [file pone.0289272.s002.png]

TIC of +MRM (1544 pairs): from Sample 2 (MWJDA21069\_mix01\_P) of MWJDA-21-069\_9\_JS4500-3\_C13\_MWDB4.0\_LSQ\_20220110.wiff (Tur...

Max. 2.7e7 cps.

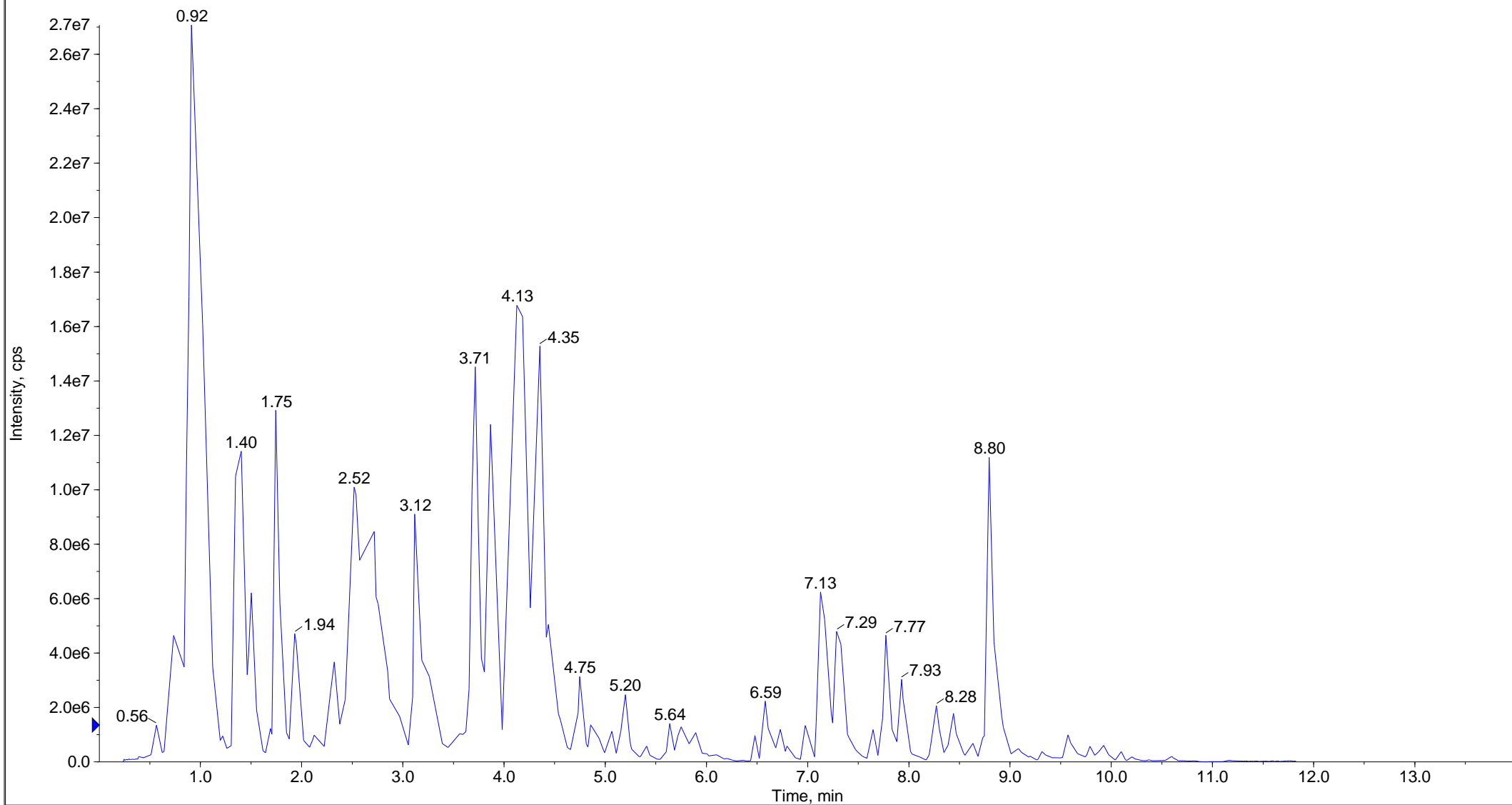

Supplement: S3 Fig — (PDF) [file pone.0289272.s003.pdf]

■ XIC of -MRM (1448 pairs): 463.088/300.000 amu Expected RT: 3.7 ID: mws0061 from Sample 17 (MWJDA21069\_mix01\_N) of MWJDA-21-069\_...

Max. 1.0e6 cps.

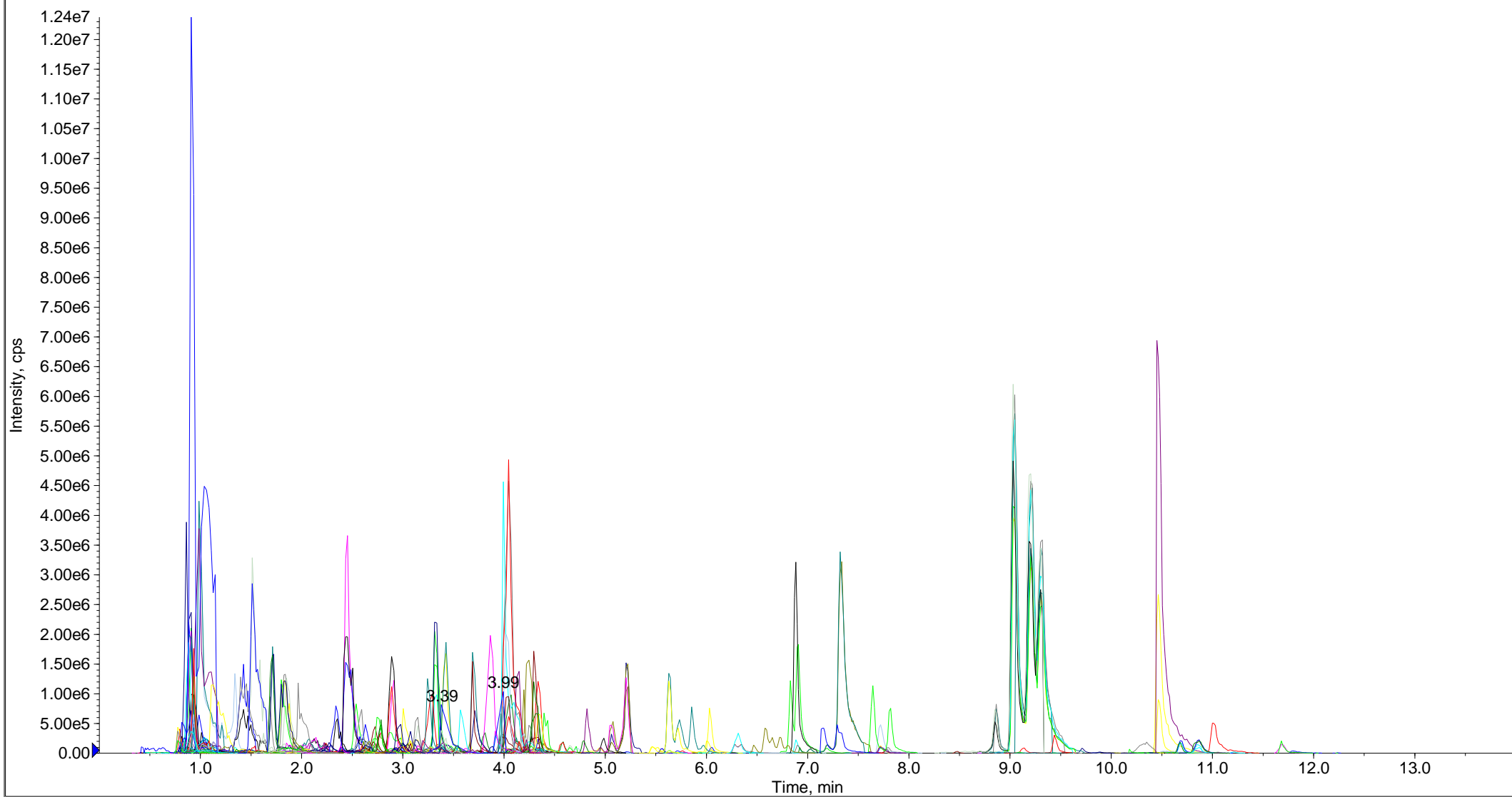

Supplement: S4 Fig — (PDF) [file pone.0289272.s004.pdf]

■ XIC of +MRM (1544 pairs): 465.139/303.090 amu Expected RT: 4.3 ID: Lmzp002365 from Sample 2 (MWJDA21069\_mix01\_P) of MWJDA-21-06...

Max. 5.3e5 cps.

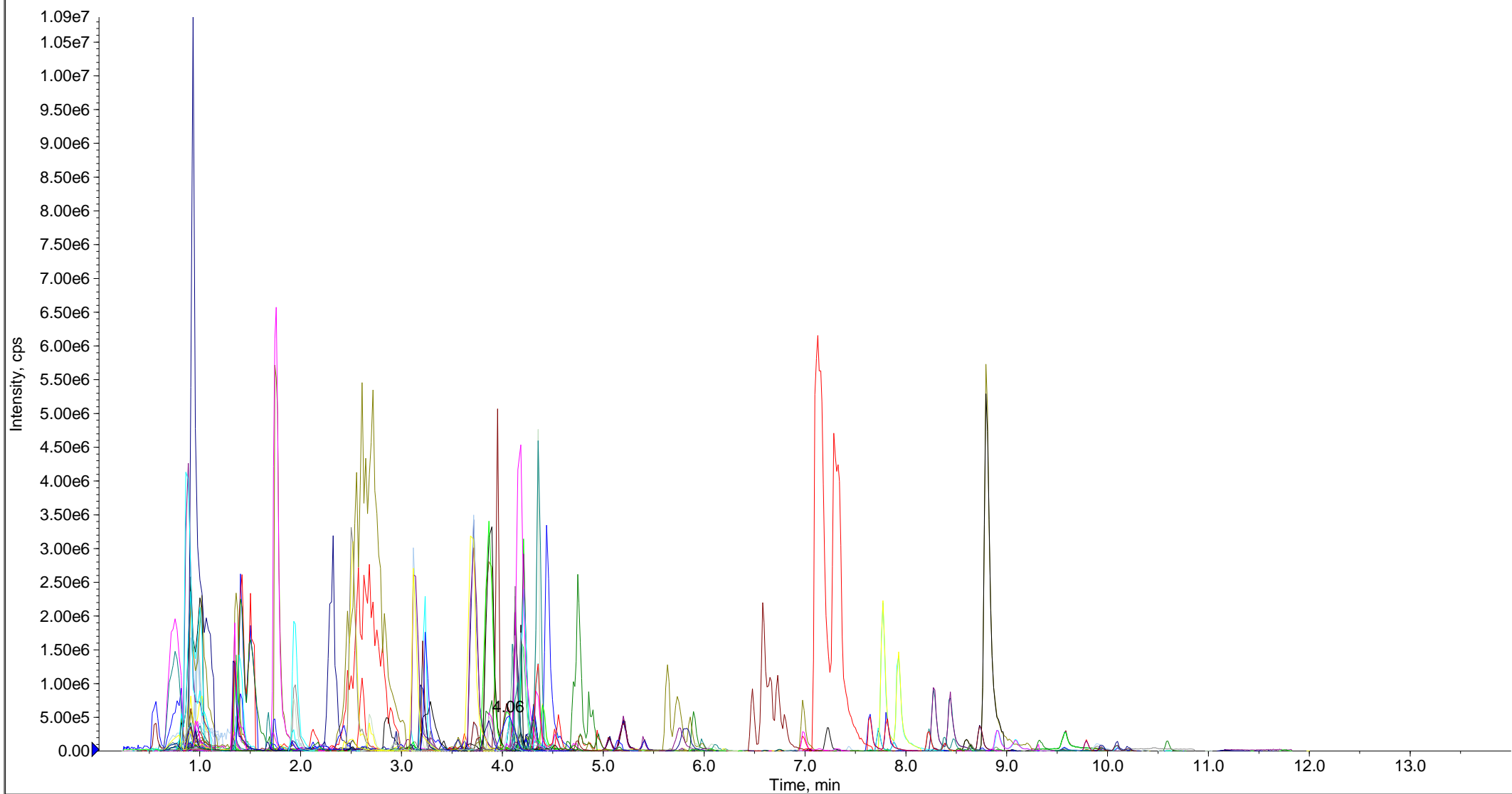

Supplement: S5 Fig — (PDF) [file pone.0289272.s005.pdf]

■ TIC of -MRM (1448 pairs): from Sample 33 (MWJDA21069\_mix01\_N) of MWJDA-21-069\_9\_JS4500-3\_C13\_MWDB4.0\_LSQ\_20220110.wiff (Tu...

Max. 4.6e7 cps.

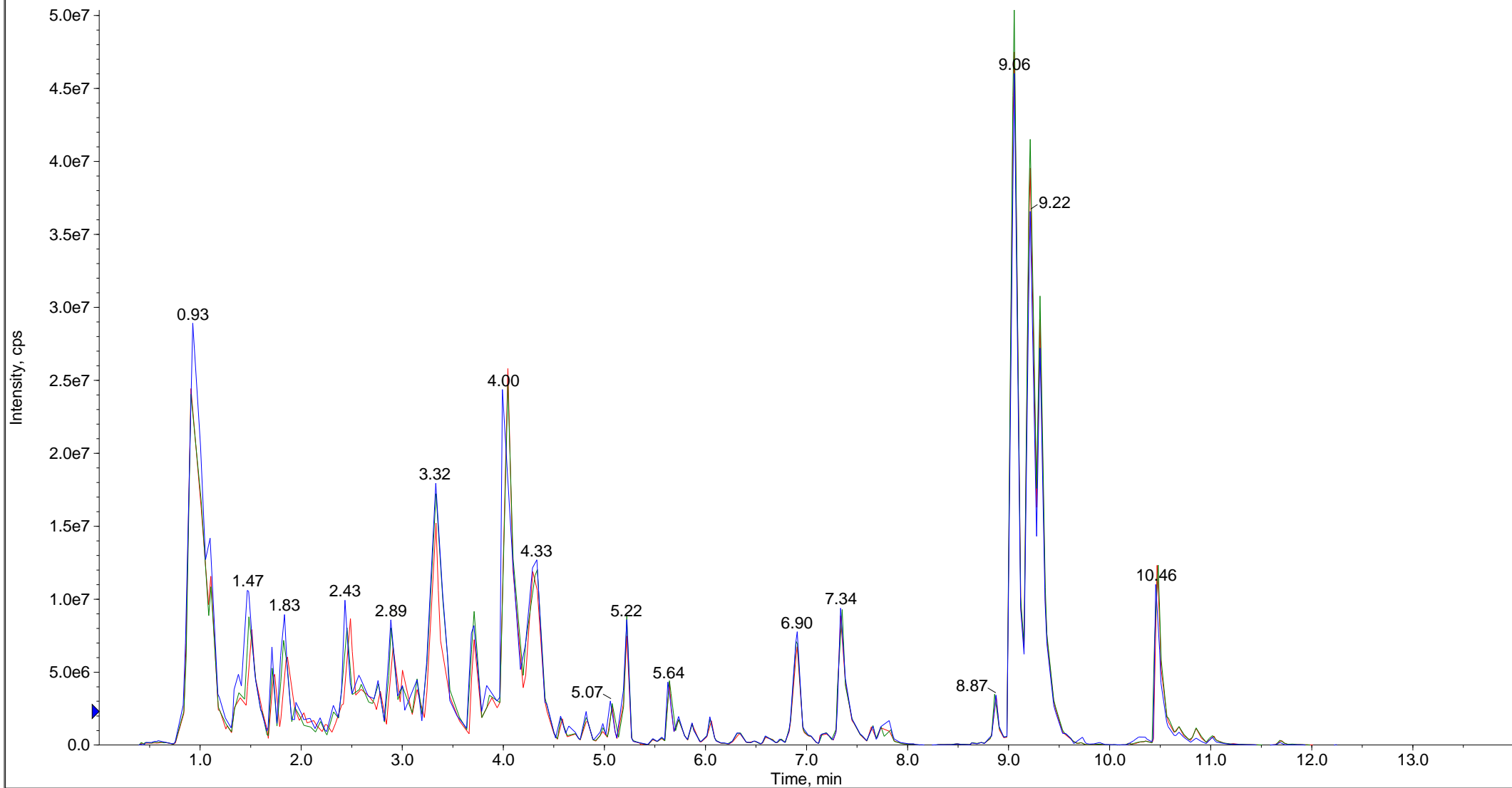

Supplement: S6 Fig — (PDF) [file pone.0289272.s006.pdf]

■ TIC of +MRM (1544 pairs): from Sample 2 (MWJDA21069\_mix01\_P) of MWJDA-21-069\_9\_JS4500-3\_C13\_MWDB4.0\_LSQ\_20220110.wiff (Tur...

Max. 2.7e7 cps.

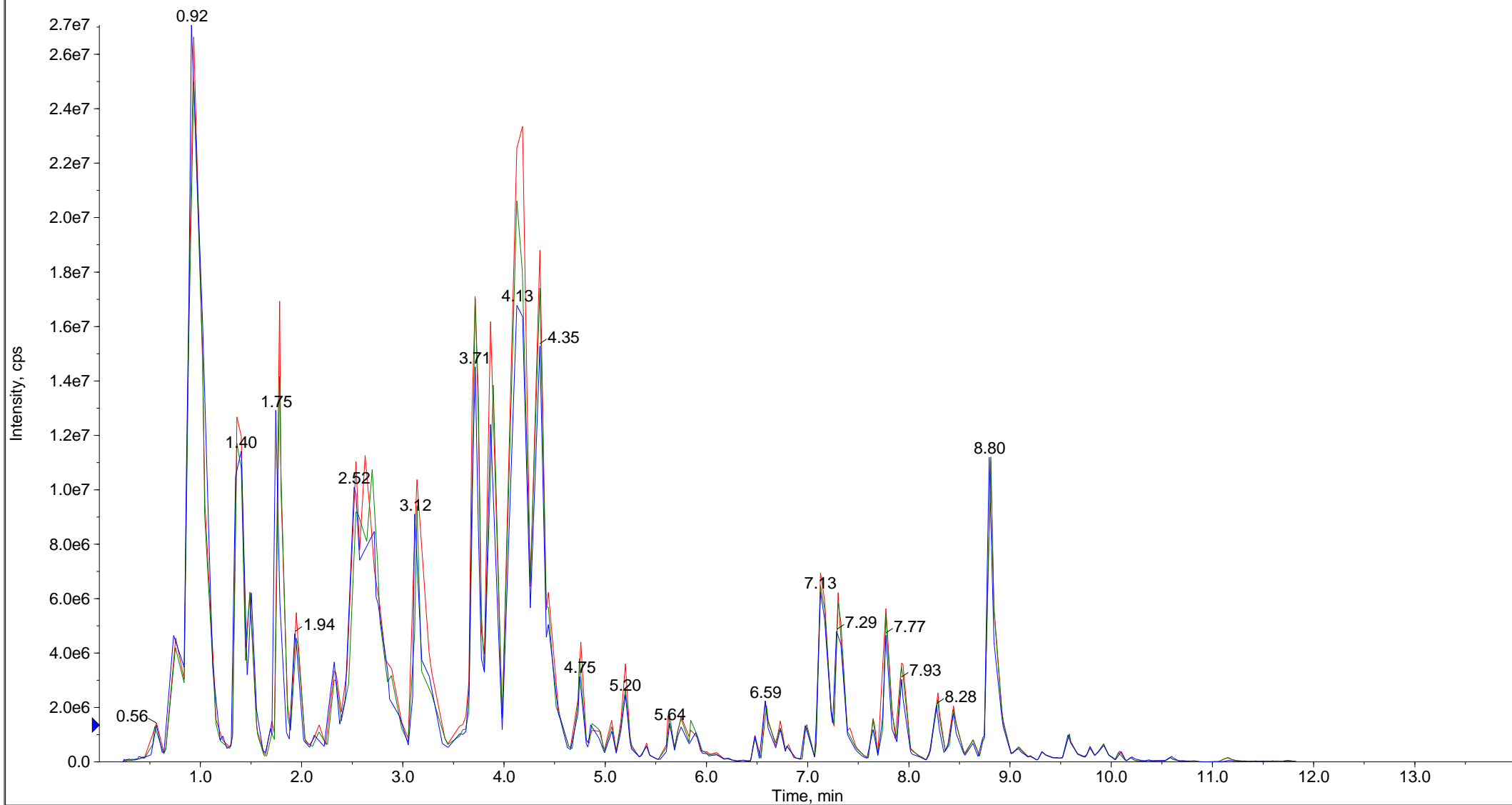

Supplement: S7 Fig — (PDF) [file pone.0289272.s007.pdf]

TIC of -MRM (1448 pairs): from Sample 17 (MWJDA21069\_mix01\_N) of MWJDA-21-069\_9\_JS4500-3\_C13\_MWDB4.0\_LSQ\_20220110.wiff (Tu...

Max. 3.5e7 cps.

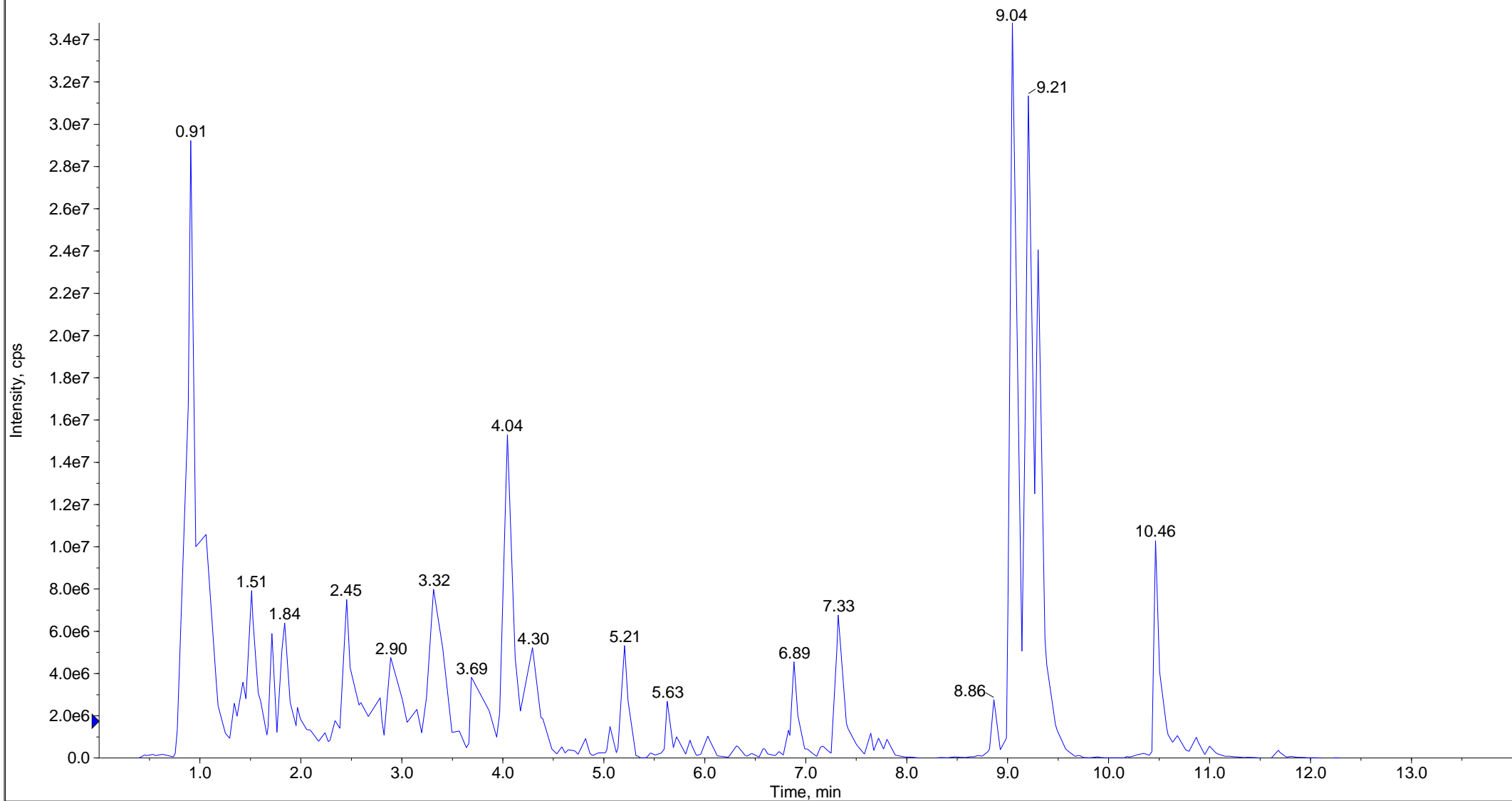

Supplement: S8 Fig — (PDF) [file pone.0289272.s008.pdf]
